# Effect of Gazpacho, Hummus and Ajoblanco on Satiety and Appetite in Adult Humans: A Randomised Crossover Study

**DOI:** 10.3390/foods10030606

**Published:** 2021-03-12

**Authors:** David Planes-Muñoz, Carmen Frontela-Saseta, Gaspar Ros-Berruezo, Rubén López-Nicolás

**Affiliations:** Food Science and Human Nutrition Department, Faculty of Veterinary, Regional Campus of International Excellence “Campus Mare Nostrum”, Campus de Espinardo, University of Murcia, 30100 Murcia, Spain; david.planes@um.es (D.P.-M.); gros@um.es (G.R.-B.); rubenln@um.es (R.L.-N.)

**Keywords:** appetite, satiety, VAS, gazpacho, hummus, ajoblanco

## Abstract

Nowadays, overweight and obesity has reached an epidemic level around the world. With the aim to tackle them, an interesting strategy is the study of food and ingredients with satiety properties. In addition to reducing food and/or calorie intake, this type of foods must be included as part of a healthy diet. With regard to this, it is well known that the Mediterranean Diet (MD) is a feeding pattern that helps us to maintain good health, providing an adequate intake of micronutrients and active compounds. With this background, the main aim of this research was to identify MD foods with a high satiating potential capacity. For this purpose, three typical foods of the Mediterranean region, mainly based on vegetables, were selected: hummus, ajoblanco and gazpacho. As a control, white bread was used. Twenty-four human healthy volunteers consumed a standard breakfast followed by the different typical Mediterranean foods, and then the subjective sensation of hunger and satiety for each food was assessed by visual analogue scales (VAS) during 3 h. Subsequently, volunteers had ad libitum access to a standard meal. The results indicate that gazpacho showed the highest satiating scores, despite the fact that it was not the food that provided the highest protein or fibre amount. More studies of this type are needed to determine the proportion and/or combination of ingredients from these classical Mediterranean recipes that could enhance human satiety.

## 1. Introduction

Nowadays, enhanced satiety foods, ingredients or nutrients are being widely studied [1,2,3]. Different studies indicate that the acquisition of knowledge in this area could facilitate the design of diets or foods to help us with reducing appetite. Thus, it could be a good strategy in the fight against overweight and obesity, since it could reduce both food and calories intake. This strategy on human satiety could help to reduce overweight and obesity prevalence and associated health complications, such as cardiovascular disease, diabetes, musculoskeletal disorders and some type of cancers [4,5]. With regard to this, different nutrients and food components included in a Mediterranean Diet (MD) have been studied to analyse their satiety value and thus help us in the control of food intake. Previous studies have focused on the study of fibre and macronutrients such as proteins, carbohydrates and fats, concluding that proteins and fibre are the most satiating nutrients [6,7]. Moreover, some studies have also focused on the possible effect of micronutrients on appetite, obtaining very promising results [8]. However, to date, our knowledge about the role of micronutrients is limited. With regard to this, foods included in a MD are mostly vegetables that, when frequently consumed, provide adequate amounts of most minerals and vitamins and also provide beneficial active compounds. Typical foods from this type of MD diet could provide a control calorie intake and, with healthy properties, could be a good choice in weight management. For this reason, considering both potential satiety properties and nutritional composition of typical Mediterranean foods, four different foods widely consumed in the Mediterranean region were chosen: hummus, ajoblanco, gazpacho and white bread [9,10,11]. Moreover, sensory properties of foods and palatability were also measured since they also play a key role in food control intake. Flavour and/or palatable texture can strongly determine the amount of ingested food [12]. Taking this background into account, the aim of this study was to identify the satiety feelings and appetite control capacity of different foods included in the MD.

## 2. Materials and Methods

### 2.1. Sample Description

Ajoblanco, gazpacho, hummus, all consisting of blended vegetables, and white bread were chosen to study their effect on human satiety. Hummus is a soft paste made from cooked, mashed chickpeas, blended with tahini, olive oil and lemon juice providing high protein and fibre content [11]. Both nutrients are related to human satiety. Ajoblanco is a cold soup typically from the south of Spain, containing bread, almonds, garlic and olive oil. It was chosen for its almond content, as this type of nut has demonstrated promising results in other satiety studies [9]. Gazpacho is also a typical cold soup originally from the south of Spain. It is traditionally prepared with a mix of several vegetables such as tomato, cucumber, pepper, onion, garlic and olive oil. Gazpacho was included in the study due to its high fibre and micronutrients (vitamins and mineral) content [10,13,14]. White bread, made from refined wheat, which basically provides carbohydrates (which have not been described as the most satiating components) and low amounts of proteins and fibre (described as high satiating components), was used as negative control [8]. Ajoblanco, gazpacho and hummus were prepared in our facilities following the traditional recipe, while bread was supplied by a local bakery. To calculate nutrients’ content of the preload ingested by participants and to prepare the relevant figures related to nutrient content, food composition tables [15] and the Easy Diet software (Xyris software, Brisbane, Australia), supported by the Spanish Association of Dietetics and Nutrition, were used, after adjusting for moisture content.

### 2.2. Study Participants

The present study was carried out in the department of Food Science and Nutrition in the Faculty of Veterinary Sciences (University of Murcia) on 24 healthy volunteers, 9 males and 15 females, aged 30.6 ± 5.58, with body mass 62.13 ± 15.32 kg, height 167.39 ± 9.04 cm, BMI 22.09 ± 2.57 kg/m${}^{2}$, fat 22.63 ± 6.86% and muscle 21.20 ± 4.75%. The sample size was calculated using a 95% of confidence level, a variance of 7,818,770 and a maximum error of 1195.94, obtained from previous satiety using visual analogue scales (VAS) results of our research group, with a result of n=21. The final recruitment was 26 volunteers, due to the possible loss of follow-up of the study. The inclusion criteria were non-smokers, BMI between 18.5 and 24.9 kg/m${}^{2}$ and age between 18 and 55 years. None of the volunteers was a professional athlete, was pregnant, had any medical condition or was on medication. The Bioethics Committee of the University of Murcia approved the study in accordance with the ethical principles of the current Declaration of Helsinki with the code 2051/2018. All volunteers gave their written and signed informed consent before participation in the study. Recruitment of volunteers was done through posters around the university and other zones of the city of Murcia, word-of-mouth, acquaintances and e-mail contacts. Subjects did not receive any payment for their participation in the study.

### 2.3. Procedure

**Figure 1:**
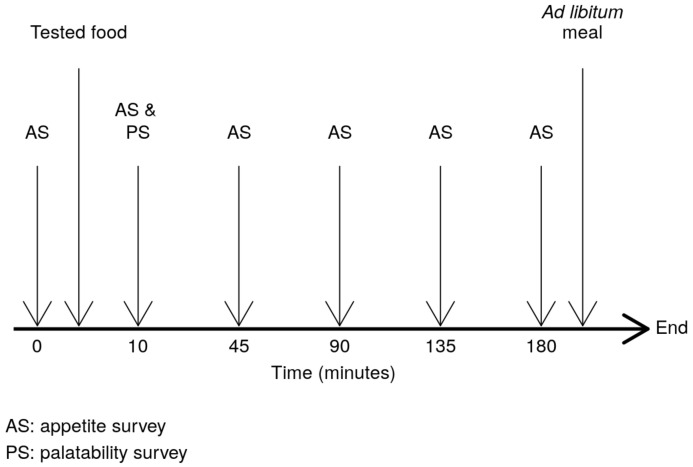
Flow diagram representing the different stages of the study design.

This work was a randomised crossover trial, registered in Open Science Framework (osf.io/7bxgz), with three different preloads (gazpacho, hummus and ajoblanco) using white bread as control [8]. The study design is presented as a flow diagram in Figure 1. Each participant arrived to our facilities on four different days (with at least one week of wash-out period), one for each analysed food. Women participating in the study did not carry out any study day during menstruation and none of the participants performed vigorous sports or drank alcohol the day before each test. Volunteers came in a fasting mode, at least since midnight the night before, each day to have breakfast around 9:00 a.m. consisting of 100 mL of black coffee (or 100 mL of green tea) with non-caloric sweetener. One hour later, volunteers filled in the first visual analogue scales (VAS) survey [16] about appetite and satiety feelings and just after that volunteers consumed the appropriate food. The description of the preload food, the water amount to match the foods’ volume and the preload foods composition is presented in Table 1.

The order in which every food was tested for every participant was calculated using the function sample of the software R. Consequently, participants had the foods in a different order across the study. The amount of ingested food was 10% of the total energy expenditure for each subject in the study, which was calculated using the Harris and Benedict formula [17]. It is one of the most commonly used equations in clinical practice, being the oldest and having undergone the most extensive validation [18]. To consider the gastric filling effect, differences in the volume among the foods assessed were matched using an appropriate amount of water when necessary. Just after volunteers consumed whole food and water, they filled in another two VAS surveys (one about appetite and another about palatability). After that, a new appetite survey was filled in every 45 min, during 3 h. The amount of water that every participant had during the first study day (from preload to ad libitum meal) was annotated to make sure that participants had the same amount of water on the remaining study days. Immediately after the last survey, an ad libitum test meal was carried out, consisting of traditional pizza “Margherita” (241 kcal/100 g) whose ingredients were dough, tomato, cheese and olive oil. Three pizzas for each test meal (≈1080 g) were baked and cut in small square pieces to prevent participants knowing the amount of served pizza. Then, the total amount of pizza was weighed and served including, in the guidelines for volunteers, that they can eat as much pizza as they wanted until they were satisfied. When the participants finished eating, the remaining pizza on the plate was weighed again to calculate the ingested amount. Every participant repeated the entire process three times, one for each remaining tested food in the study.

### 2.4. Assessment of Appetite and Palatability

Surveys used to measure both satiety and palatability were formed by visual analogue scales (VAS), which consisted of the corresponding questions, followed by a 10 cm horizontal line with the expressions “nothing at all” and “extremely” anchored at each end of the line. Participants answered surveys by marking on the line according to their feelings. The VAS survey scale used to evaluate satiety and appetite included four main questions about hunger, fullness, desire to eat and prospective consumption. It is a validated tool to measure appetite and satiety sensations [16] and widely used for this procedure [19,20,21,22]. From these surveys’ scores, areas under the curve (AUC), incremental areas under curve (ΔAUC) and incremental VAS score at 180 min (ΔVAS${}_{180}$) were calculated as described in Section 2.5. These data give us important information regarding the appetite and satiety sensations during the study. Food palatability (as another important factor on satiety regulation) was measured through a survey provided to volunteers immediately after eating the tested foods. Surveys used consisted of VAS and six questions about pleasure of eating the food, desire to eat more, sweetness, savouriness, tastiness and creaminess. To evaluate the possible caloric compensation after the tested foods, the calorie intake at each ad libitum meal was also calculated.

### 2.5. Data and Statistical Analysis

With data obtained from the VAS surveys scores, areas under curve (AUC) and incremental area under curve (ΔAUC), by subtracting the base line (time 0) from the remaining scores, were calculated by using the trapezoidal rule. In addition, from the VAS scores, the incremental score at 180 min (ΔVAS${}_{180}$) was calculated. Outliers from VAS scores were removed after determination by using multivariate imputation by chained equations with random forest machine learning method (Shah et al., 2014). Data normality were confirmed through Shapiro–Wilk test. Then, data were compared using a multilevel lineal model with preloads as predictor variable and participant ID as random effects variable, setting maximum likelihood as the method. Tukey post hoc was used to determine the differences between preloads. Robust bootstrap one-way repeated measures ANOVA for the trimmed means was used when data did not have normality or sphericity (Checked by using Maunchly’s test). The same statistical procedure was followed to analyse data obtained from palatability assays. All tests were set with a *p* value <0.05. The data and statistical analysis, as well as the figures included in this article, were made by using the language and environment for statistical computing R version 4.0.3 [23]. Two participants did not finish the study due to a change in their work schedules which did not permit them to continue, so all their data were removed from the study to avoid missing values, and, therefore, the final analysis included 24 participants.

## 3. Results

**Figure 2:**
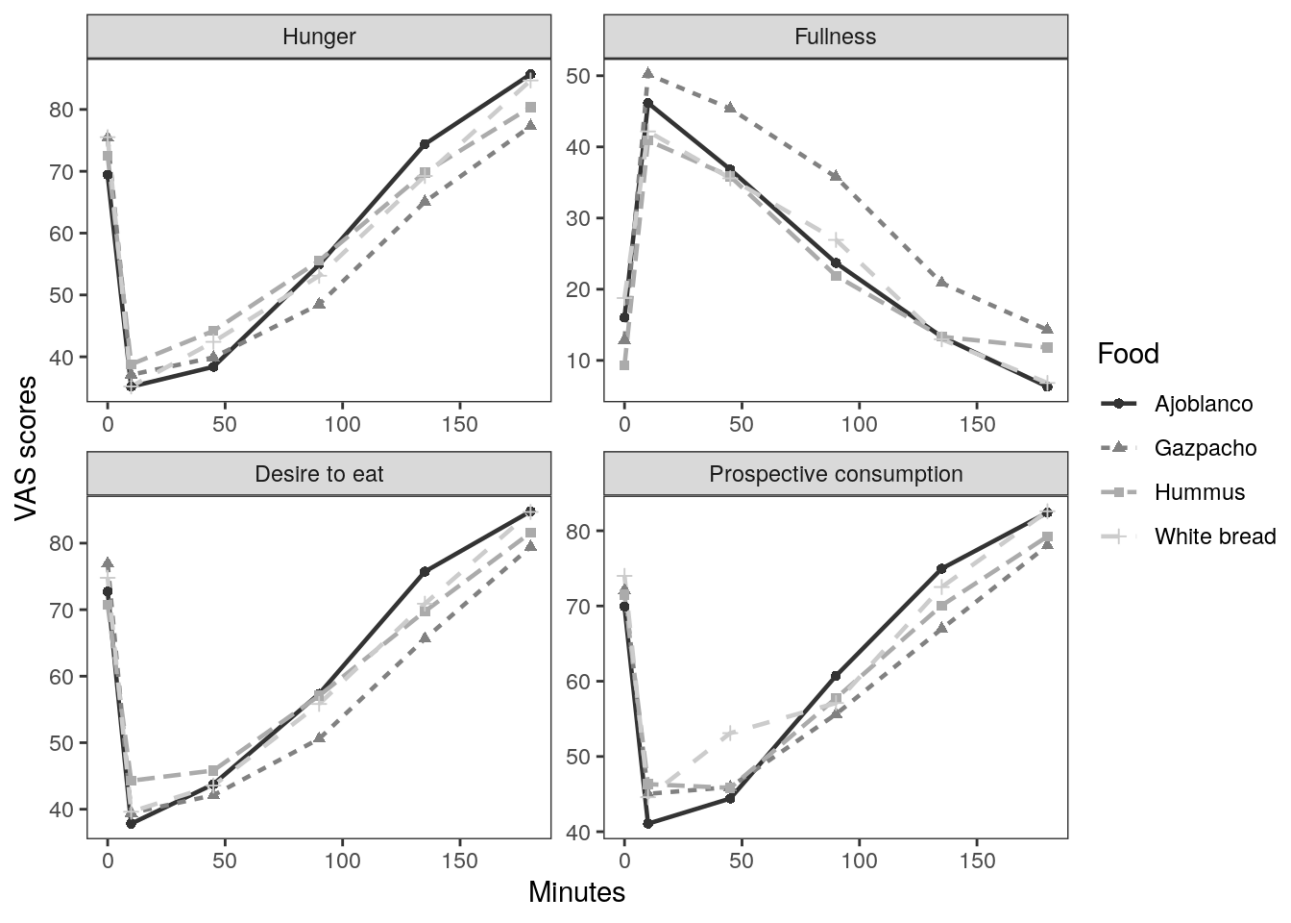
Subjective appetite scores after the four preloads test. Data are presented as mean values (*n* = 24).

The results of this study include the subsequent calorie intake in ad libitum meals, the appetite sensations obtained from the VAS ratings (Figure 2) to obtain AUC, ΔAUC and ΔVAS${}_{180}$. Moreover, palatability and nutritional composition of every tested preload were analysed.

### 3.1. Palatability Assays

**Figure 3:**
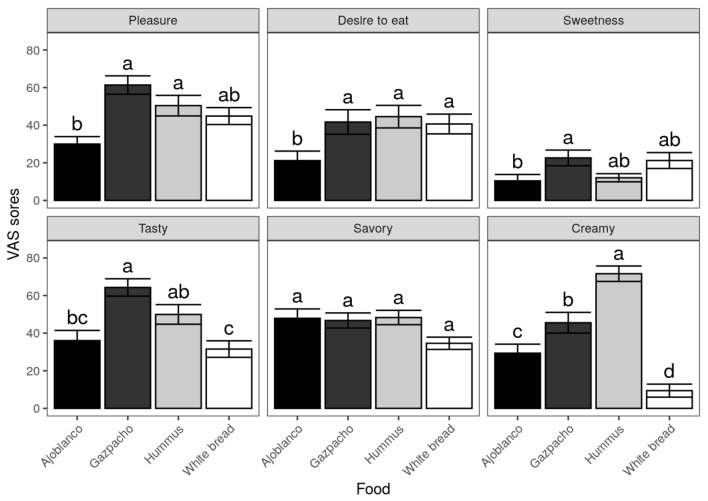
Palatability scores measured by VAS for each tested food. Data are presented as mean values ± SEM.

Another factor for food intake control is the need to consider palatability as a key factor, since more palatable foods have shown to be related to overeating, probably by a stimulation of brain circuits related to the reward responses [12]. This is the reason in the present study a palatability survey was carried out immediately after the intake of the foods under study. Participants were asked about the pleasure they felt when eating the respective food, the desire to eat more, the sweetness, the tastiness, the savouriness and the creaminess. As can be observed in Figure 3, the most pleasant foods were, in order, gazpacho, hummus, white bread and ajoblanco, with statistically significant differences (p<0.05) between gazpacho and hummus as compared to ajoblanco.

Regarding the desire to eat more of the corresponding food, ajoblanco was the worst rated, obtaining the remaining food a similar score amongst them and without significant differences. The food scored as sweeter was gazpacho, without significant differences with hummus and white bread. Ajoblanco was the lowest scored, although no differences were found with hummus and white bread. In terms of flavour, gazpacho was the best rated with statistically significant differences (p<0.05) between it and ajoblanco and white bread. Relating to creaminess, significant differences between all the studied foods were observed, being hummus the creamiest food followed by gazpacho, ajoblanco and white bread. There were no differences relating to savouriness.

### 3.2. Appetite and Satiety Sensations Analysis from the VAS Scores

Data from appetite and satiety VAS surveys related to hunger, fullness, desire to eat and prospective consumption provide an estimation of appetite and satiety in volunteers during the study time and the results can be observed in Table 2. According to other studies [24,25,26], white bread was used as reference food compared to the other foods assayed because of its low content of fibre and proteins, which are reported as key components of food intake control.

As can be observed in Table 2, AUC, ΔAUC and ΔVAS${}_{180}$ of gazpacho showed the best satiating mean values for hunger, fullness, desire to eat and prospective consumption, followed, in most cases, by hummus. Statistical analysis showed no significant differences (p<0.05) among hummus, ajoblanco and white bread in any studied case. For AUC data, gazpacho gave the greatest fullness sensation (p<0.05) compared with the remaining foods. In the case of ΔAUC, participants reported less hunger sensations and more fullness sensations for gazpacho, compared with ajoblanco and white bread (p<0.05), without significant differences between hummus and the remaining preloads. Hunger for ΔVAS${}_{180}$ showed gazpacho as the food inducing the most significant hunger suppression among the four assessed preloads. Fullness sensations in this case showed gazpacho as the most satiating food (p<0.05) but without differences with hummus.

### 3.3. Ad Libitum Test Meal

Three hours after the corresponding food intake, volunteers had an ad libitum meal to assess the possible caloric intake compensation. A large amount of pizza “Margherita” (241 kcal/100 g) was baked, weighed and then served to participants on each test day. When participant finished eating, the remaining amount of pizza was weighed to calculate the grams and caloric intake. There were no statistically significant differences in the amount of pizza eaten after ingestion of foods under study. Nevertheless, the same trend obtained from VAS results was observed in the data from the test meal, showing that volunteers ate less pizza when they had gazpacho as preload. The amount ingested by participants, represented as a mean ± SEM, were 509.24 ± 178.9 g (1227.27 ± 431.0 kcal) after eating white bread, 502 ± 177 g (1210 ± 427 kcal) after eating ajoblanco, 492 ± 169 g (1187 ± 408 kcal) after eating hummus and 480 ± 172 g (1156 ± 414 kcal) after eating gazpacho.

### 3.4. Nutritional Composition of the Preloads

**Figure 4:**
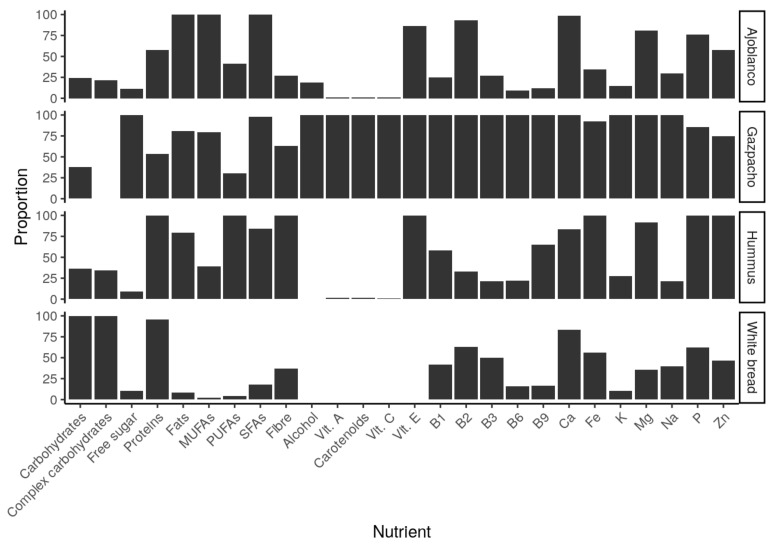
Percentage of nutrients provided by the different tested foods in relation to the maximum quantity of each nutrient.

The nutritional contribution of the tested preloads was calculated, as commented in Section 2.1, and the obtained results are set out below. In Figure 4, the total average quantity that volunteers ingested is represented through their nutrient content, being shown as a percentage of the highest amount of the four tested foods and, therefore, the food contributing the greatest amount of each nutrient can be easily observed.

**Figure 5:**
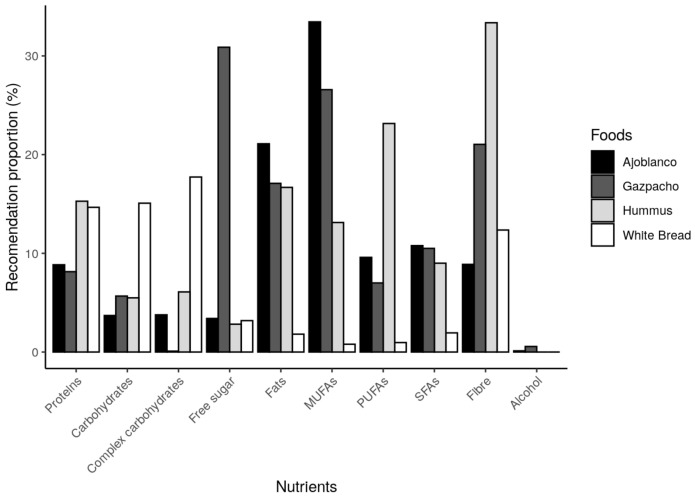
Percentage of macronutrients, fibre and alcohol provided by the ingested amount of each tested food, represented as a percentage of the general intake recommendations

**Figure 6:**
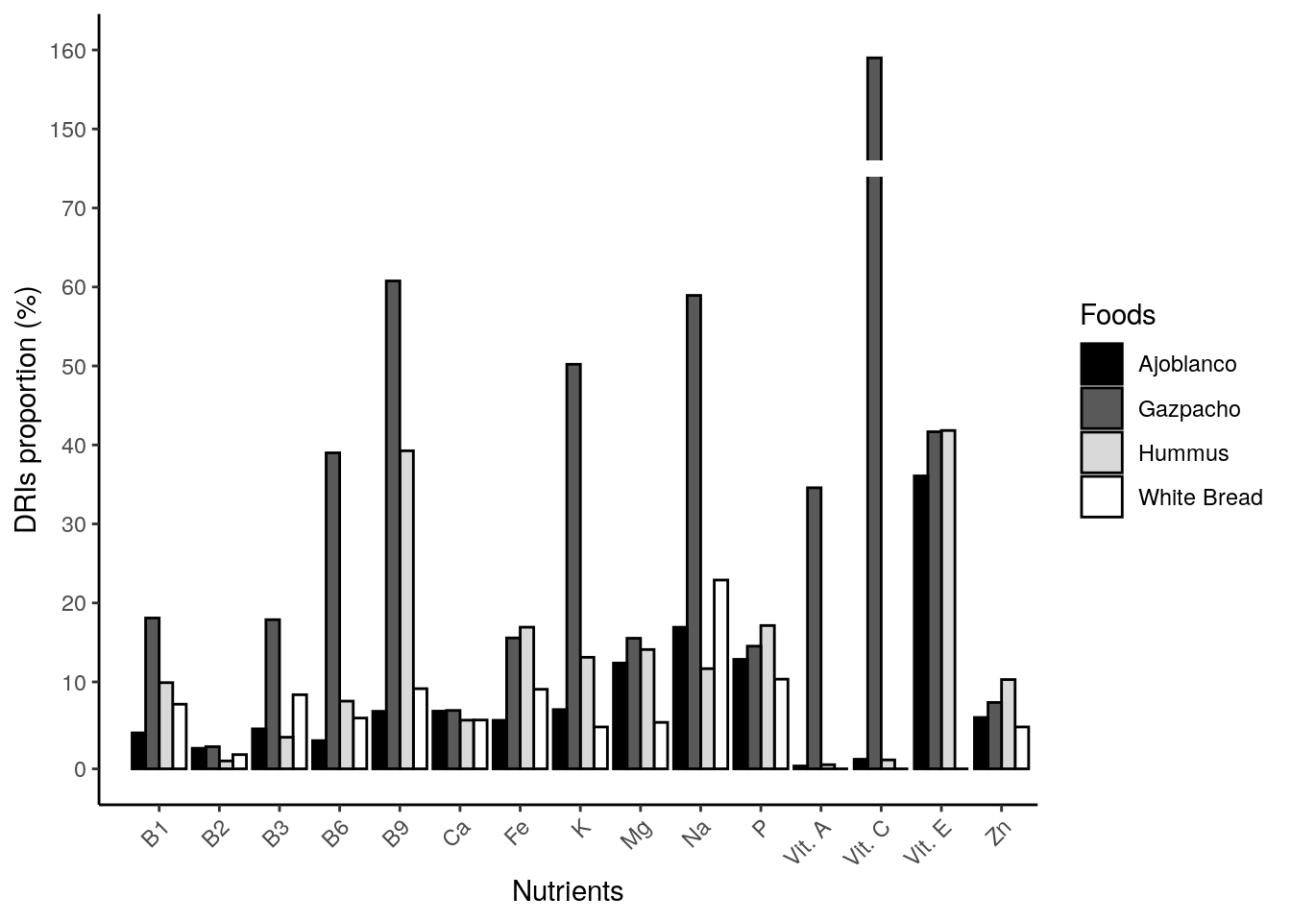
Percentage of vitamins and minerals provided by the ingested amount of each tested food, represented as a percentage of the dietary reference intakes (DRI) for each micronutrient.

As can be observed in Figure 4, gazpacho shows the highest amount of free sugar, alcohol and most micronutrients. This cold tomato soup is the second tested food in iron, phosphorus and zinc content. Therefore, it can be suggested that gazpacho provides the highest amount of free sugar and most micronutrients by calories, compared to the other three studied foods. In Figure 5 and Figure 6, macro- and micronutrients contents can be observed. The results are expressed as a percentage of the Daily Reference Intake (DRI) [27] or general recommendation about each nutrient or substance.

Hummus provided the highest amount of proteins, fibre, polyunsaturated fatty acids (PUFAs), P, Zn and Fe, while ajoblanco provided the largest amount of fats (except for PUFAs). The highest carbohydrate contribution was observed in white bread, which also contributed the second highest amount of protein. Focusing again on gazpacho, low quantities of proteins, total and complex carbohydrates and PUFAs were observed, whilst providing the largest amount of free sugar, compared with the other three tested foods. Nevertheless, as Figure 6 shows, in accordance with Figure 4, gazpacho contained the highest DRIs proportion in almost all represented micro-nutrients, being also the second highest assessed food in the other minerals.

The contribution of carotenoids regarding a general intake recommendation [28] was also calculated. The results show that gazpacho is the food that contributes the largest amount of the compound followed by humus and ajoblanco, with all providing more than the recommend intake.

## 4. Discussion

It can be deduced from results observed from the palatability VAS surveys that gazpacho seems to be the most palatable food for volunteers followed by hummus, which, as commented above, could increase the appetite. However, these two foods (gazpacho and hummus) also were the ones that showed the best satiety results through the VAS survey, compared to ajoblanco and white bread Therefore, it seems that the effects on appetite regulation found in this study could be due to different factors and not only palatability.

Results referring to appetite and satiety sensations, obtained from VAS surveys, seem to indicate that, among the four foods tested, gazpacho was the most satiating one in this study. It is important to consider that these kinds of studies usually focus on the satiating effect of the macronutrients (protein, fats and carbohydrates) to control hunger. In this regard, it is accepted that, among macronutrients, proteins induce the highest hunger suppression, followed by complex carbohydrates and fats, respectively [8]. In addition, dietary fibre (non-digestible carbohydrates) in foods could play an important role in hunger control [3]. However, relating to nutrient content according to recipes of tested foods, gazpacho does not contain the highest amount of either proteins or fibre.

Gazpacho induced the greatest appetite suppression measured by VAS, and it does not seem to be due to caloric content or ingested volume because the volunteer’s intake consisted of the same number of calories and volume (matched with water). Regarding this, different weights for the different assessed foods were used with the aim of providing the same energy density in all of them. Different satiety studies add water to food to decrease its energy density because satiety is highly affected by the number of calories. Moreover, it has been reported that drinking water as a beverage together with a meal had a similar effect on satiety as that incorporating an equivalent amount of water into the food [29]. In addition, isocaloric foods with different volumes affect satiety (higher volume causes a lower food intake) suggesting a role of energy density in the overconsumption of foods [30].

As Figure 5 shows, although gazpacho has the highest amount of alcohol, this amount comprises a very small percentage of maximum daily recommendation (less than 1%) and, in any case, alcohol was reported to increase appetite and reduce satiety [31]. With this background, alcohol should not be considered as causing the satiating effect observed with gazpacho. Regarding the food composition, the most satiating food in this study (gazpacho) stands out for its high contribution of free sugar, vitamins, minerals and other non-nutrient substances such as carotenoids. It is important to consider that these compounds can be modified, dissolved and released during the digestion process and thus could modify satiety signalling in different ways when compared with non-digested foods, which could explain differences in satiety response [32]. This deserves more research to elucidate the effect of digestion on the satiety capacity of foods. Although current knowledge seems to indicate that, regarding food composition, proteins and fibre show the highest satiating capacity [6,33], this effect cannot be confirmed in the present study. Among tested foods, hummus contained the largest amount of protein and fibre, while gazpacho showed the lowest protein content and was second in fibre but demonstrated the most satiating potential. At this point, it is also important to note that the satiating capacity of the different compounds included in foods is not quite clear [34]. In the case of proteins, after digestion, satiety feelings can be modified in different ways because it is dependent upon peptide transporters or different technological processes among other factors. Knowledge of the effect of digested protein released during digestion process on satiety is still waiting more research [35]. In the case of high fibre foods, digestion seems to increase satiety [32]; however, it is probably that other food components generated by breakdown during digestion can also modify satiety feelings.

A plausible explanation about satiety effects observed in this study is the glucostatic theory [36], which was proposed by Jean Mayer in 1955, and it still stands today [37]. According to that, glucose receptors in the hypothalamus could detect changes in blood glucose that can regulate appetite and feelings of satiety. In this way, the higher glycaemic levels could induce higher satiety sensations, and vice versa. It is also worth noting that a high fibre content can slow down the absorption of these sugars, being a factor that enhances medium-term satiety [38]. However, in accordance with our results, and as can be observed in Figure 5, hummus was the food with the highest amount of fibre. Gazpacho provided the greatest amount of free sugars, but not the greatest amount of total carbohydrates, which eventually reach the blood as glucose as well. When comparing the glycaemic index (GI) of these foods, it can be observed that both gazpacho and hummus have a similarly low GI [39,40]. In addition, white bread has been classified as a high GI food [41], and it does not seem that differences observed in satiation can be attributed only to the amount of sugar. The satiety and hunger sensations ratings measured by VAS through the study time can be observed in Figure 2. With regard to hunger, desire to eat and prospective consumption, results up to 45 min were similar among the different tested foods, but later on gazpacho scores showed a higher satiety effect. In the case of fullness, gazpacho scores showed the greatest values during the 3 h of study. Hummus showed the second greatest VAS scores on satiety, but this effect could be observed at 180 min, later than in the case of gazpacho. Therefore, the presence of higher amount of free sugar in gazpacho seems not to be of high importance since, as mentioned above, the GI of hummus and gazpacho were similar. However, the results indicate that gazpacho is more satiating, compared to other foods, as time goes on. Therefore, it is not clear that the highest satiety observed in gazpacho effect could be attributed to the carbohydrate’s contribution. As can be observed in Figure 6, gazpacho provides the greatest contribution of vitamins and minerals. Although less studied, some researches have observed that micronutrients can play an important role in appetite regulation and weight management [8,42]. In this regard, the most studied micronutrient is calcium and in vivo studies in rats showed that following a low calcium diet, the animals chose a high calcium beverage [8], suggesting a mechanism to mitigate a calcium deficit. Moreover, a calcium and multivitamin supplemented diet has shown to be an appetite control [43] and produced less hunger sensations [44] in human studies. However, there are few studies on the satiety effect of food micronutrients’ content. A possible explanation for these findings could point to a compensatory mechanism regarding nutritional status of some micronutrients [42], but the real cause is still unknown. Among the four studied foods in this work, gazpacho contributed, in general terms, the largest quantity of most micronutrients (Figure 4 and Figure 6). However, the study design did not include a positive control for vitamins and mineral, so the satiety effect observed for this food cannot be attributed to this factor.

With regard to results obtained from the ad libitum test meal, a similar trend was observed when compared to VAS results, but no significant differences were found. It might be due to an excessive time between the preload and ad libitum test meal, since, after 180 min, the possible effects on appetite and intake may have disappeared. However, we selected 3 h because our aim was to evaluate the satiety in medium-long term, since previous studies suggested that satiety differences were manifested after 2 h. Another possible flaw of the study design, regarding the ad libitum test meal, could be the choice of food offered for the test. Analysing the data carefully, the mean calories pizza intake by the participants in this study was almost half (47%) of the total calorie intake that these volunteers need in a day (2501 Kcal), when the regular intake at lunch is around 30% of the daily calories. It possible that a popular and palatable food, such as pizza, offered ad libitum, could lead participants to overeating.

## 5. Conclusions

This study was carried out to determine the satiating effect on humans of different Mediterranean foods. Gazpacho, hummus and ajoblanco were selected for their satiety potential effect according to their nutritional composition. As a control, white bread was used. The satiating effect was evaluated through VAS surveys and ad libitum test meal. VAS surveys results show gazpacho as the most satiating food, despite the fact that it did not contain the highest amount of proteins, fibre or carbohydrates, compounds that have been shown to have a satiating effect. These results suggest a possible role of micronutrients on satiety. Therefore, more studies in this regard are necessary to clarify these effects. Regarding the ad libitum test meal, the same trend was observed, but without statistical differences among the tested foods, possibly due to an excessive time between the preloads and the ad libitum test meal or to the selected food for the test.

## Figures and Tables

**Table 1:** Description of the preloads tested in the present study.

	Ajoblanco	Hummus	Gazpacho	White Bread
Grams	261.6	93.4	500.4	88.3
Water (g)	238.7	407.0	0.0	412.1
Kcal	208.1	208.1	208.1	208.1
Fats (g)	16.5	13.0	13.3	1.4
Carbohydrates (g)	10.2	15.1	17.9	41.5
Simple sugar (g)	1.3	0.0	15.0	1.8
Fibre (g)	2.2	8.3	5.3	3.7
Proteins (g)	4.8	7.6	4.1	7.3

The amount of water indicated in this table refers to the amount of water that the participants drank along with the different foods, in order to balance the volumes of the preloads consumed.

**Table 2:** Visual analogue scales (VAS) results expressed as AUC, ΔAUC and ΔVAS score at 180 min for each analysed food.

Foods	Hunger	Fullness	Desire to Eat	Prospective Consumption
Area under curve (AUC)				
Ajoblanco	10,512 ± 552 ${}^{a|\alpha }$	4320 ± 493 ${}^{b|\beta }$**	11,073 ± 569 ${}^{a|\beta }$	11,122 ± 588 ${}^{a|\alpha }$
Gazpacho	9656 ± 791 ${}^{a|\alpha }$	5878 ± 660 ${}^{a|\beta }$**	9973 ± 836 ${}^{a|\beta }$	10,483 ± 809 ${}^{a|\alpha }$
Hummus	10525 ± 657 ${}^{a|\alpha }$	4216 ± 476 ${}^{b|\beta }$**	10,722 ± 708 ${}^{a|\beta }$	10,786 ± 670 ${}^{a|\alpha }$
White bread	10,277 ± 696 ${}^{a|\alpha }$	4415 ± 508 ${}^{b|\beta }$**	10,616 ± 653 ${}^{a|\beta }$	11,188 ± 548 ${}^{a|\alpha }$
Incremental area under curve (ΔAUC)				
Ajoblanco	−2081 ± 851 ${}^{a|\alpha }$*	1557 ± 421 ${}^{b|\beta }$*	−2347 ± 773 ${}^{a|\beta }$	−1577 ± 901 ${}^{a|\beta }$
Gazpacho	−4082 ± 666 ${}^{b|\alpha }$*	3474 ± 635 ${}^{a|\beta }$*	−3966 ± 806 ${}^{a|\beta }$	−2526 ± 456 ${}^{a|\beta }$
Hummus	−2766 ± 790 ${}^{ab|\alpha }$*	2578 ± 549 ${}^{ab|\beta }$*	−2334 ± 736 ${}^{a|\beta }$	−2261 ± 608 ${}^{a|\beta }$
White bread	−3157 ± 723 ${}^{ab|\alpha }$*	1426 ± 635 ${}^{b|\beta }$*	−2837 ± 741 ${}^{a|\beta }$	−2218 ± 652 ${}^{a|\beta }$
Incremental VAS${}_{180}$ score (ΔVAS${}_{180}$) (mm)				
Ajoblanco	16 ± 6 ${}^{a|\alpha }$*	−7 ± 3 ${}^{b|\beta }$*	8 ± 4 ${}^{a|\beta }$	11 ± 5 ${}^{a|\alpha }$
Gazpacho	1 ± 3 ${}^{b|\alpha }$*	2 ± 2 ${}^{a|\beta }$*	2 ± 3 ${}^{a|\beta }$	4 ± 3 ${}^{a|\alpha }$
Hummus	8 ± 4 ${}^{a|\alpha }$*	−1 ± 1 ${}^{ab|\beta }$*	11 ± 4 ${}^{a|\beta }$	8 ± 3 ${}^{a|\alpha }$
White bread	11 ± 3 ${}^{a|\alpha }$*	−7 ± 2 ${}^{b|\beta }$*	9 ± 3 ${}^{a|\beta }$	6 ± 3 ${}^{a|\alpha }$

Data are represented as mean values ± SEM. Within each measure (AUC, ΔAUC and ΔVAS_180_) in the same column, different letters (a, b) denote statistically significant differences. * *p* < 0.05 (values are 0.018 in Fullness ΔAUC, 0.047 in Fullness ΔVAS_180_, 0.043 in Hunger ΔVAS_180_ and 0.048 in Hunger ΔAUC); ** *p* < 0.01 (values are 0.002). Greek letters (*α*–*β*) denote large, medium and small general effect sizes, respectively.

## Data Availability

The data presented in this study are available on request from the corresponding author.

## Abbreviations

The following abbreviations are used in this manuscript:

AUC	Area under curve
BMI	Body mass index
MD	Mediterranean Diet
PUFA	Polyunsaturated fatty acid
VAS	Visual analogue scales
ΔAUC	Incremental area under curve
ΔVAS${}_{180}$	Incremental VAS score at 180 min
